# Engineered Lubricative Lecithin-Based Electrospun Nanofibers for the Prevention of Postoperative Abdominal Adhesion

**DOI:** 10.3390/pharmaceutics16121562

**Published:** 2024-12-06

**Authors:** Junhan Li, Hao Lin, Jinghua Li, Yi Wang

**Affiliations:** 1 Basic Research Key Laboratory of General Surgery for Digital Medicine, Affiliated Hospital of Hebei University, Baoding 071000, China; lijunhan5@163.com; 2 College of Mechanical & Energy Engineering, Beijing University of Technology, Beijing 100124, China; haolin0104@emails.bjut.edu.cn

**Keywords:** electrospun, nanofibers, hydration lubrication, abdominal adhesion

## Abstract

**Background**: Postoperative abdominal adhesion is a prevalent complication following abdominal surgery, with the incidence of adhesion reaching up to 90%, which may precipitate a range of adverse outcomes. Although fibrous membranes loaded with various anti-inflammatory or other drugs have been proposed for anti-adhesion, most of them suffer from drug-induced adverse effects. **Methods**: In this study, a lecithin-based electrospun polylactic acid (PLA) nanofibrous membrane (L/P-NM) was developed for the prevention of postoperative abdominal adhesion, utilizing the hydration lubrication theory. The loaded zwitterionic lecithin allows the nanofiber surface to strongly bind water molecules to create a hydration lubrication interface. **Results**: As the TGA results show, the content of bound water in the nanofibers increased significantly with the increase in the lecithin content. Tribological test results show that L/P-NM reached a minimum coefficient of friction (COF) of about 0.112. Additionally, the developed nanofibrous membranes possess favorable tensile property and biocompatibility. Rat postoperative abdominal adhesion model evaluation results demonstrated that L/P-NM possesses significant anti-adhesive performance, with an adhesion score of only 1. **Conclusions**: Therefore, this study offers a promising strategy for efficiently preventing abdominal adhesion.

## 1. Introduction

Postoperative abdominal adhesion is a prevalent complication following abdominal surgery, with the incidence of adhesion reaching up to 90% [1]. These adhesions can lead to a range of detrimental outcomes, including bowel obstruction [2], chronic pain [3], female infertility [4], and recurrent surgical interventions [5]. Despite the use of adhesion lysis, abdominal trauma during the procedure typically results in recurrent adhesion for over 80% of patients [6]. Therefore, the prevention of abdominal adhesion formation is a more effective solution than adhesion lysis, helping in patient recovery, reducing pain, and decreasing reoperation rates [7].

In clinical practice, deploying a physical barrier material at the site of injury is a prevalent strategy for preventing postoperative abdominal adhesions [8]. Such materials isolate the injured peritoneum and prevent the formation of fibrous tissue bonds between the damaged peritoneum and adjacent tissues, thus preventing the occurrence of postoperative abdominal adhesions [9]. Among the various physical barrier materials, electrospun nanofibrous membranes have been widely studied and applied due to their ease of functionalization, favorable biodegradability, and robust mechanical properties [10]. For instance, while electrospun polylactic acid (PLA) membranes have been employed in clinical settings, their efficacy in preventing adhesions is limited, as the hydrophobic surface of PLA nanofibrous promotes cell adhesion [11]. Certain fibrous membranes that incorporate anti-inflammatory agents can prevent abdominal adhesions by suppressing inflammation [12,13,14,15]. For example, Bahareh Kheilnezhad et al. [16] developed a polycaprolactone (PCL)/polyethylene glycol (PEG) nanofibrous membrane enriched with ibuprofen, which leveraged ibuprofen’s anti-inflammatory attributes to prevent abdominal adhesion effectively. However, the side effects associated with drugs raised safety concerns and are not conducive to clinical translational applications [17]. Previously, we reported the development of the SLNM, which was based on the hydration lubrication theory [18]. According to the hydration lubrication principle, the charged groups of zwitterion on the fibrous membrane surface attract surrounding water molecules, forming a hydration layer. This hydration layer can decrease the friction coefficient on the fibrous membrane surface, thereby preventing cellular adherence and postoperative adhesion [19]. Nevertheless, the preparation of the SLNM requires monomer 2-Methacryloyloxy ethyl phosphorylcholine (MPC) to undergo a free radical polymerization reaction on the fibrous membrane surface, a process that produces various by-products [20]. These by-products elevate the safety risks of the material [21], posing challenges to its clinical translation. Therefore, there is an urgent need to develop a fibrous membrane with high biosafety for the prevention of abdominal adhesions, which will have great potential for translational clinical applications.

Lecithin, a primary constituent of cell membranes, plays a crucial role in upholding the integrity and stability of these structures. It is extensively present in egg yolks and across a range of plant and animal tissues [22,23]. The U.S. Food and Drug Administration (FDA) has classified lecithin as a GRAS (Generally Recognized as Safe) substance [24], and it is extensively utilized as an ingredient in foods, offering good biosafety [25,26]. In addition, lecithin is a natural zwitterion with polar groups in its molecular structure that can attract water molecules to form a hydration layer [27]. PLA is a synthetic biodegradable polymer with good biocompatibility and biodegradability, which has been approved by the FDA for clinical use as a medical material [28]. In the present study, lecithin-based electrospun nanofibrous membranes were developed utilizing the hydration lubrication theory to ameliorate postoperative abdominal adhesions. These membranes were synthesized by blending lecithin and PLA for the purpose of making them electrospun. Lecithin creates a hydrated lubrication layer on the surface of the nanofibrous membrane, preventing postoperative abdominal adhesions [29,30]. Therefore, this study offers a promising strategy for efficiently preventing abdominal adhesion.

## 2. Materials and Methods

### 2.1. Material Preparation

**Figure 1:**
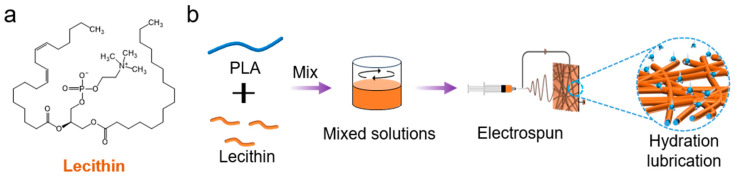

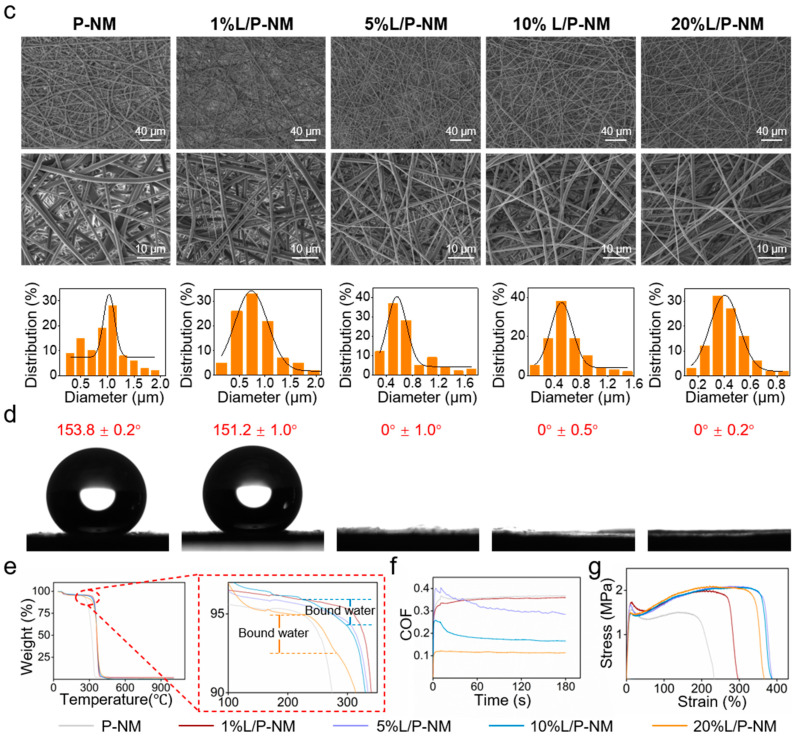
Material characterization: (**a**) Chemical structural formula of lecithin; (**b**) Schematic of the preparation method and hydration lubrication of lecithin-lubricated fibrous membrane; (**c**) SEM image and diameter distribution of nanofibrous membrane; (**d**) water contact angle; (**e**) thermogravimetric analysis curve; (**f**) friction test curve; (**g**) tensile test curve.

PLA (molecular weight: 87,000), lecithin, and other chemicals were acquired from Beijing Solaibao Technology Co., Ltd. (Beijing, China). As shown in Figure 1a, a solution of hexafluoroisopropanol containing PLA (20%, *w*/*v* to HFIP) and lecithin (1–20%, *w*/*w* to PLA) was electrospun into fibrous membranes (L/P-NM), and the spinning parameters were a voltage of 20 kV, a collection distance of 18 cm, a feed rate of 6 mL/h, and room temperature. The L/P-NM was then vacuum-dried for 3 days to remove the residual solvent.

### 2.2. Material Characterization Methods

The surface morphology of the fibrous membrane samples was analyzed using SEM. Pt was sputtered on the samples by a vacuum ion sputtering unit (QuorumSC7620, Quorum, Lewes, UK) for 10 min, and then the samples were randomly observed using a scanning electron microscope (SEM, TESCAN MIRA LMS, TESCAN, Brno, Czech Republic) and the images were acquired using Thermo Scientific Maps software 3.0. The fibrous membranes’ surface wettability, content of components, lubrication performance, and mechanical properties were characterized using water contact angle measurements, thermogravimetric analysis, tribological assessments, and tensile strength evaluations, respectively. A contact angle analyzer with (OCA-20, Dataphysics Instruments, Filderstadt, Germany) was used to place a 5 μL drop of deionized water onto the surface of the sample, and the Water contact angle (WCA) values of the membranes were measured after the droplets had stabilized, with assistance from Shiyanjia Lab (www.shiyanjia.com, accessed on 26 November 2024). A thermogravimetric analysis measurement was conducted using the simultaneous thermal analyzer (Hitachi STA200, Tokyo, Japan) in a nitrogen atmosphere (temperature range: room temperature—1000 °C, temperature rise: 10 °C/min) to obtain the thermogravimetric curve of the membranes. Tribological tests were performed using UMT-5 (Bruker Nano Inc., Berlin, Germany) in rotational mode (normal load: 0.5 N, radius of rotation: 3 mm, speed: 50 mm·min^−1^, lubricant medium: SBF, temperature: 37 °C). Membrane samples were used as lower specimens, firmly bonded to the upper surface of the slide with 3M tape, and slid against the upper specimen (SiN ball of 6 mm diameter). Fibrous membranes cut into 5 mm × 50 mm strips were subjected to tensile testing using a universal mechanical testing machine (HS-1000A, Shanghai HESON Instrument Technology, Shanghai, China) to obtain stress–strain curves.

### 2.3. In Vitro Test of Anti-Cell Adhesion

The anti-cell adhesion property of the fibrous membranes was assessed using NIH/3T3 fibroblasts. The fibrous membrane samples were placed in a 24-well culture plate via ultraviolet light for 24 h. Using the slides of the cells as a control, a cell suspension with a density of 4 × 10^4^ cells per well was inoculated onto the surface of the samples and incubated under conditions of 37 °C and 5% CO_2_. Cytoskeletal staining was performed on days 1 and 3, and the images were observed using a laser scanning confocal microscope (LSM-800, Zeiss, Oberkochen, Germany). The data were collected using ZEN software (version 2.3).

### 2.4. In Vivo Degradation Test

The biodegradability of the fibrous membrane material was evaluated by implanting the fibrous membrane samples into the abdominal cavity of SD rats. The rats were anesthetized with sodium pentobarbital, an incision was made in the abdominal area, and 20% L/P-NM was implanted on the inner wall of the rat’s abdominal cavity. The incision was then sutured, and the samples were removed 28 days later. Afterward, they were dried in an oven at 37 °C, and the surface morphology of the fibrous membrane was observed using SEM.

### 2.5. Evaluation of Cytocompatibility

The cell compatibility of the fibrous membrane was detected using the CCK-8 method. First, the fibrous membranes were cut into strips of 1 cm × 2 cm and soaked in 5 mL of the cell culture medium at 37 °C for overnight incubation. After removal, the fibrous membranes were filtered to obtain the corresponding extract. The NIH/3T3 fibroblasts were seeded into 96-well plates (2 × 10^3^ cells/well) and incubated at 37 °C and 5% CO_2_. After adhering to the plate, the culture medium was replaced with the extract for cultivation. The cells were incubated for 6 h, 12 h, and 24 h, respectively, after which the cytotoxicity kit (CCK-8) was used to detect them. The optical density (OD) value of the solution at 450 nm was tested using a microplate reader.

### 2.6. Evaluation of Blood Compatibility

The fibrous membranes from each group were cut into strips measuring 1 cm × 4 cm and then submerged in 10 mL of physiological saline solution and incubated at 37 °C overnight. After removing the fibrous membranes, the extracts were filtered to obtain the corresponding extracts. To the extracts, 0.1 mL of diluted rat anticoagulated plasma was added and mixed, with distilled water serving as the positive control and physiological saline solution as the negative control. The solutions from each group were then incubated at 37 °C for 1 h, followed by centrifugation at 3500 rpm for 5 min. The supernatant was taken, and the OD value of the solution at 450 nm was tested using a microplate reader.

### 2.7. In Vivo Anti-Adhesion Test

The in vitro anti-adhesive properties of the fibrous membrane were investigated using initial and recurrent abdominal adhesion models in rats. Male Sprague-Dawley (SD) rats, weighing 200 ± 25 g, were acquired from Beijing SPFT Biotechnology Co., Ltd. (Beijing, China). Both experimental adhesion models randomly allocated the rats into four groups, each containing six rats: the control group, the P-NM group, the 20%L/P-NM group, and the Normal group (untreated, used for organ toxicity comparison).

#### 2.7.1. Rat Abdominal Initial Adhesion Model

In the rat abdominal initial adhesion model, the rats were anesthetized with sodium pentobarbital. The sterile gauze was used to scrape the exposed cecum and corresponding abdominal wall until bleeding occurred. Subsequently, a 1 cm × 2 cm fibrous membrane was implanted to cover the damaged site, securing it to the abdominal wall. The cecum was returned to the abdominal cavity and fixed against the injured site of the abdominal wall. Thereafter, the incision was sutured sequentially. Postoperatively, the rats were killed on the 14th day to obtain the tissue–membrane composite material, as well as the major organs, including the heart, liver, spleen, lung, and kidney. The evaluation was based on the gross observations, adhesion score, H&E staining, Masson staining, immunofluorescence staining, and organ index (organ index = organ weight × 100/body weight).

#### 2.7.2. Rat Abdominal Recurrent Adhesion Model

In the rat abdominal recurrent adhesion model, the rats were anesthetized with sodium pentobarbital. The sterile gauze was used to scrape the exposed cecum and corresponding abdominal wall until bleeding occurred. The cecum was then returned to the abdominal cavity and fixed at the site of the abdominal wall injury. Subsequently, the incision was sutured layer by layer. After surgery, a second operation took place on the 7th day to remove adhesions, followed by the implantation of a 1 cm × 2 cm fibrous membrane over the injured area, which was sutured to the abdominal wall. The cecum was then returned to the abdominal cavity and fixed against the injured site of the abdominal wall. Finally, the incision was sutured sequentially. Postoperatively, on the 7th day, the rats were killed to obtain the tissue–membrane composite material and the main organs, such as the heart, liver, spleen, lung, and kidney. These were evaluated based on the gross observations, adhesion score, H&E staining, Masson staining, immunofluorescence staining, and organ indexes (organ index = organ weight × 100/body weight).

## 3. Results and Discussion

### 3.1. Material Characterization

In order to observe microscopic features, Figure 1b presents the SEM images of nanofibrous membranes incorporated with varying concentrations of lecithin (L/P-NM) and the statistical data of the fibrous diameter distribution. The results indicate that as the lecithin content increases, the fibrous diameter gradually decreases. WCA was used to evaluate the hydrophilicity/hydrophobicity of the samples, as shown in Figure 1d, with P-NM having a WCA of 153.8° ± 0.2°, 1% L/P-NM having a WCA of 151.2° ± 1.0°, and the water contact angles of L/P-NM with 5%, 10%, and 20% lecithin contents all being 0°. Because lecithin is a naturally occurring zwitterionic substance containing hydrophilic groups, this makes the fibrous membrane surface ultrahydrophilic. The results of the thermogravimetric analysis (TGA) are shown in Figure 1e, with a significant increase in the bound water content in the nanofibrous membranes from 1% to 20% lecithin content. This shows that the hydration layer on the surface of the nanofibrous membranes can be controlled by adjusting the lecithin content, and the content of bound water in the L/P-NM is closely related to its hydration lubrication behavior. In order to evaluate the tribological performance of the nanofibrous membranes, we performed friction tests, the results of which are shown in Figure 1f. The coefficient of friction (COF) values of the fibrous membrane samples decreased with an increasing lecithin content, with the 20% L/P-NM reaching a minimum COF of approximately 0.112, indicating that the incorporation of lecithin improves the lubricity of the nanofibrous membranes. Moreover, the tensile properties of the nanofibrous membranes are shown in Figure 1g. Compared to P-NM, all the L/P-NM samples exhibited higher tensile strength and elongation at break, indicating that L/P-NM possesses superior tensile properties.

### 3.2. Evaluation of Anti-Adhesion Properties In Vitro

**Figure 2:**
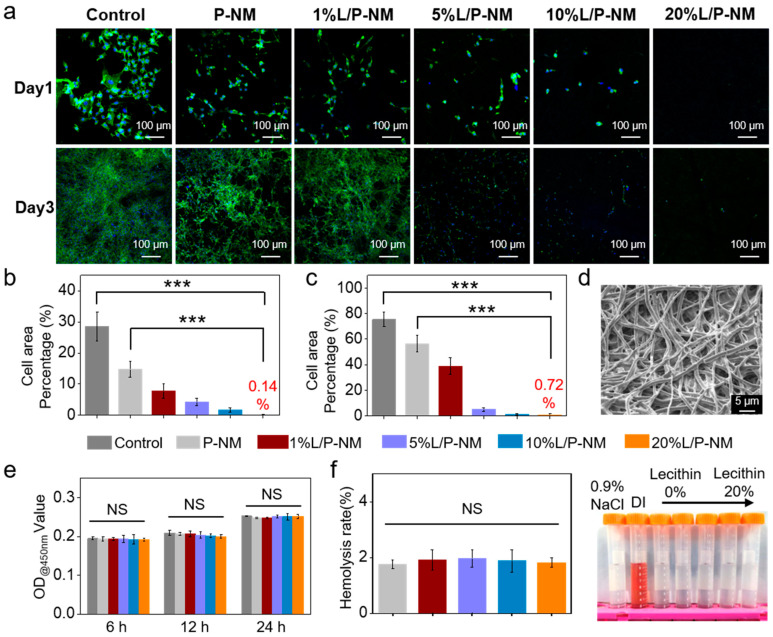
In vitro anti-adhesion and biocompatibility evaluation of L/P-NM: (**a**) Confocal images of in vitro anti-cell adhesion assay. Green color represents cytoskeleton and blue color represents the nucleus of cell; (**b**) Percentage of cell area for day 1; (**c**) Percentage of cell area for day 3; (**d**) SEM image of 20% fibrous membrane after in vivo degradation; (**e**) Cytocompatibility evaluation; (**f**) Hemocompatibility evaluation. *** *p* < 0.001, NS: no significance.

The occurrence of abdominal adhesion is closely related to the adhesion of fibroblasts, and the adhesion of fibroblasts on the surface of anti-adhesion membrane materials may lead to the occurrence of adhesions [31,32,33]. Therefore, we inoculated NIH/3T3 fibroblasts on the surfaces of various groups of nanofibrous membranes to evaluate their anti-adhesion properties in vitro. The results of cell cytoskeleton staining are shown in Figure 2a. With the increase in lecithin content, the percentage of the cell area adhering to the nanofibrous membrane surface gradually decreases (Figure 2b,c), which is due to the lecithin forming a hydration layer on the surface of the fibrous membranes, thereby blocking the adhesion of fibroblasts, demonstrating that L/P-NM has good anti-adhesion properties in vitro.

### 3.3. Evaluation of Biodegradability

L/P-NM is composed of polylactic acid and lecithin, where lecithin is a small molecule and polylactic acid is a degradable polyester material, which can be enzymatically degraded in the body into non-toxic substances such as H_2_O and CO_2_ [34,35]. The SEM image in Figure 2d shows that, after being implanted for 28 days, some fibers in 20% of the L/P-NM have fractured, indicating that the fibrous film has undergone degradation in the body.

### 3.4. Evaluation of Biocompatibility

Biocompatibility is an essential evaluation criterion for implantable medical devices or biomaterials developed for clinical translation [36]. We tested the cytotoxicity of the fibrous membrane samples in each group using the CCK-8 method, as shown in Figure 2e. The OD values in the control group cultured with a normal medium were not significantly different from those in the L/P-NM groups at all time points, indicating that L/P-NM has no significant cytotoxicity. In the in vitro hemocompatibility experiments, the hemolysis rates in the P-NM and the L/P-NM groups were less than 5% (Figure 2f), indicating the good hemocompatibility of L/P-NM. Therefore, the L/P-NM developed in this study demonstrates good biocompatibility.

### 3.5. Evaluation of In Vivo Anti-Adhesion Effect

**Figure 3:**
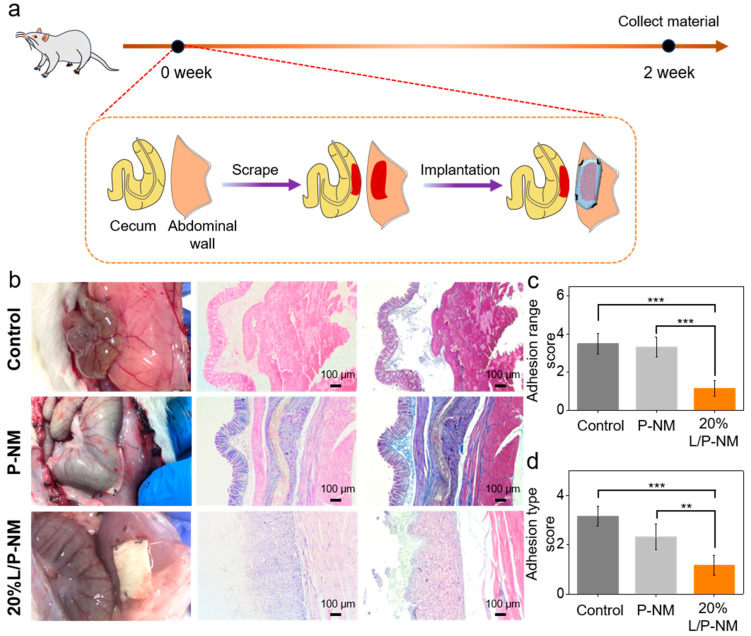
In vivo evaluation of the effect of preventing initial adhesion: (**a**) Schematic diagram of the experimental method; (**b**) Macroscopic observation and H&E and Masson staining of tissues at two weeks after surgery; (**c**) Adhesion range score; (**d**) Adhesion type score. *** *p* < 0.001, ** *p* < 0.01, *n* = 6 per group.

To evaluate the efficacy of L/P-NM in preventing postoperative abdominal adhesion in vivo, we conducted experiments using a rat abdominal initial adhesion model, as shown in Figure 3a. Fibrous membranes were applied to the damaged area to prevent the occurrence of abdominal adhesion. Adhesions typically commence within 3 to 7 days post-injury or surgery. At days 7 to 30, the gradual vascularization and collagen deposition strengthen the fibrotic bands, leading to undesirable tissue adhesion. Standardized animal models have described that 14 days is the best end point to evaluate the severity of adhesion formation [37,38]. After two weeks, the abdominal adhesion was evaluated, as shown in Figure 3b. The control group shows obvious adhesion between the cecum and abdominal wall with a wide range of adhesion; the adhesion tissue was opaque and contained obvious new blood vessels, and blunt separation was difficult. The P-NM group shows obvious adhesion between the cecum and the fibrous membranes, with the adhesion range covering almost the entire fibrous membranes; the adhesion tissue was semi-transparent, with no obvious vessels visible, and could be easily separated by blunt dissection. The 20% L/P-NM group demonstrates no adhesion between the cecum and the fibrous membranes, with only a small amount of transparent fibrous tissue attached to the surface of the fibrous membranes. H&E staining and Masson staining of tissues show (Figure 3b) that obvious adhesion could be observed between the cecum and the abdominal wall or fibrous membranes in the control and P-NM groups, while no adhesion tissue was observed in the 20% L/P-NM group. The adhesion score assessed using the Diamond method demonstrated that the adhesion range score and adhesion type score in the 20% L/P-NM group were significantly lower than those in the control and P-NM groups (Figure 3c,d) [39], indicating a good adhesion prevention effect. This effect is due to the hydrophilic lubricating layer formed on the surface of the fibrous membranes, which can prevent adhesion by resisting the adhesion of fibroblasts.

**Figure 4:**
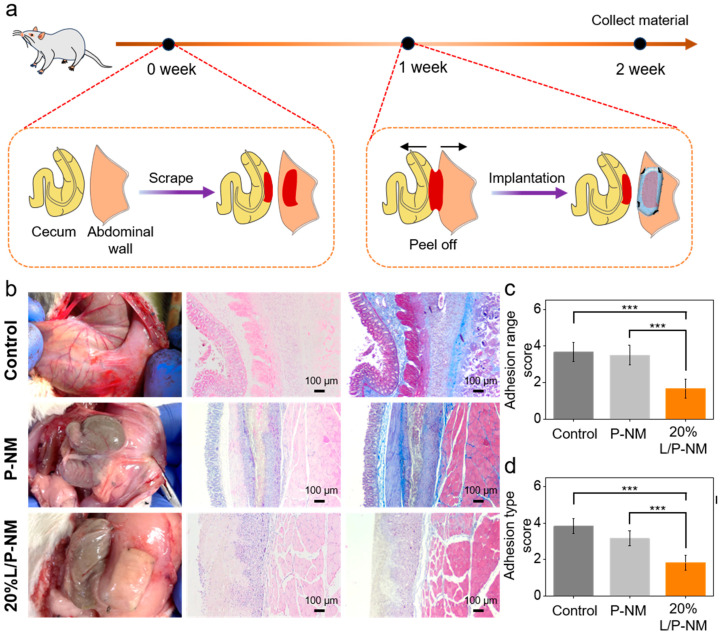
In vivo evaluation of the effect of preventing recurrent adhesion: (**a**) Schematic diagram of the experimental method; (**b**) Macroscopic observation and H&E and Masson staining of tissues at two weeks after surgery; (**c**) Adhesion range score; (**d**) Adhesion type score. *** *p* < 0.001, *n* = 6 per group.

To evaluate the preventive effects of L/P-NM on recurrent abdominal adhesion, as shown in Figure 4a, after the adhesion in the rat abdominal cavity was relieved for one week, a fibrous membrane was covered over the injured area to prevent the occurrence of abdominal adhesion. After one week, the abdominal adhesion was evaluated, as shown in Figure 4b. In the control group, there was significant adhesion between the cecum and the abdominal wall, with a wide range of adhesion; the adhesion tissues were opaque and contained a large number of new blood vessels; blunt dissection was difficult and caused considerable bleeding. The P-NM group shows obvious adhesion between the cecum and the fibrous membranes, with the adhesion range covering almost all of the fibrous membrane; the adhesion tissue was opaque and contained obvious new blood vessels, and blunt separation was difficult. The 20% L/P-NM group demonstrates no adhesion between the cecum and the fibrous membranes, with only a small amount of transparent fibrous tissue attached to the surface of the fibrous membranes. H&E staining and Masson staining of tissues showed (Figure 4b) that obvious adhesion could be observed between the cecum and the abdominal wall or fibrous membranes in the control and P-NM groups, while no adhesion tissue was observed in the 20% L/P-NM group. The adhesion score assessed using the Diamond method demonstrated that the adhesion range score and adhesion type score in the 20% L/P-NM group were significantly lower than those in the control and P-NM groups (Figure 4c,d), indicating a good adhesion prevention effect. This effect is due to the hydrophilic lubricating layer formed on the surface of the fibrous membranes, which can prevent adhesion by resisting the adhesion of fibroblasts. All experimental results were consistent with the rat model of initial abdominal adhesions, which indicates that 20% L/P-NM also has a good preventive effect on recurrent abdominal adhesions.

**Figure 5:**
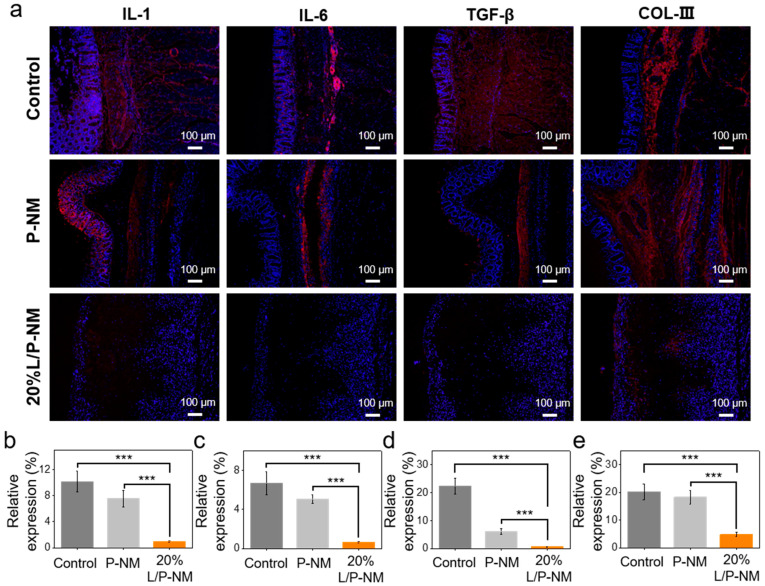
Evaluation of inflammatory factor and COL-III expression at the adhesion site in the rat abdominal initial adhesion model: (**a**) Immunofluorescence images of inflammatory factors and COL-III expression at the adhesive site. Red color represents inflammatory factors or COL-III and blue color represents the nucleus of cell; (**b**–**e**) Quantitative comparison of the inflammatory factor and COL-III expression at adhesion sites. *** *p* < 0.001.

**Figure 6:**
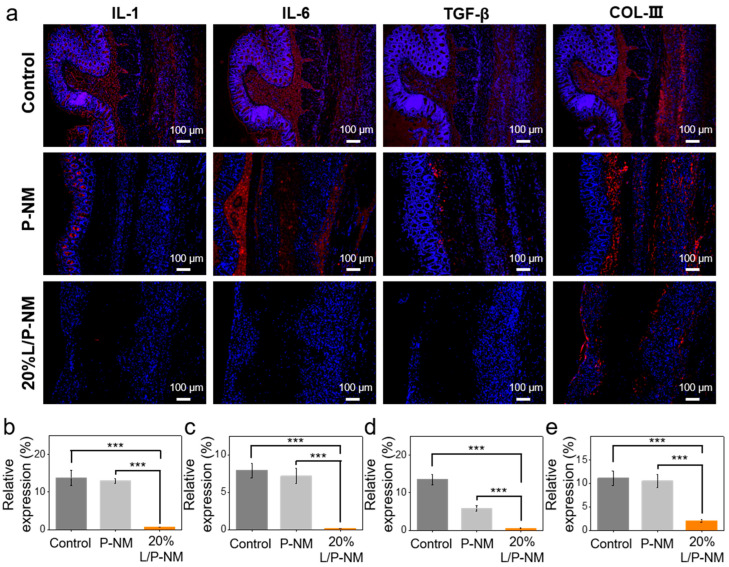
Evaluation of inflammatory factor and COL-III expression at the adhesion site in the rat abdominal recurrent adhesion model: (**a**) Immunofluorescence images of inflammatory factors and COL-III expression at the adhesive site. Red color represents inflammatory factors or COL-III and blue color represents the nucleus of cell; (**b**–**e**) Quantitative comparison of inflammatory factor and COL-III expression at adhesion sites. *** *p* < 0.001.

In the process of abdominal adhesion occurring after surgery, tissue damage leads to the disruption of abdominal integrity, increased mechanical friction between tissues, and the subsequent exacerbation of inflammation, which in turn promotes the occurrence of tissue adhesion [40,41]. Through immunofluorescence analysis, we compared the expression of inflammatory factors and COL-III in each group. The results showed that the expression of IL-1, IL-6, TGF-β, and COL-III in the 20% L/P-NM group was significantly lower than that in the control and P-NM groups (Figure 5a–e), indicating that inflammation and adhesion in the 20% L/P-NM group were milder. Similar results were also observed in the recurrent adhesion prevention experiment (Figure 6a–e). This suggests that 20% L/P-NM can reduce tissue inflammation and adhesion.

**Figure 7:**
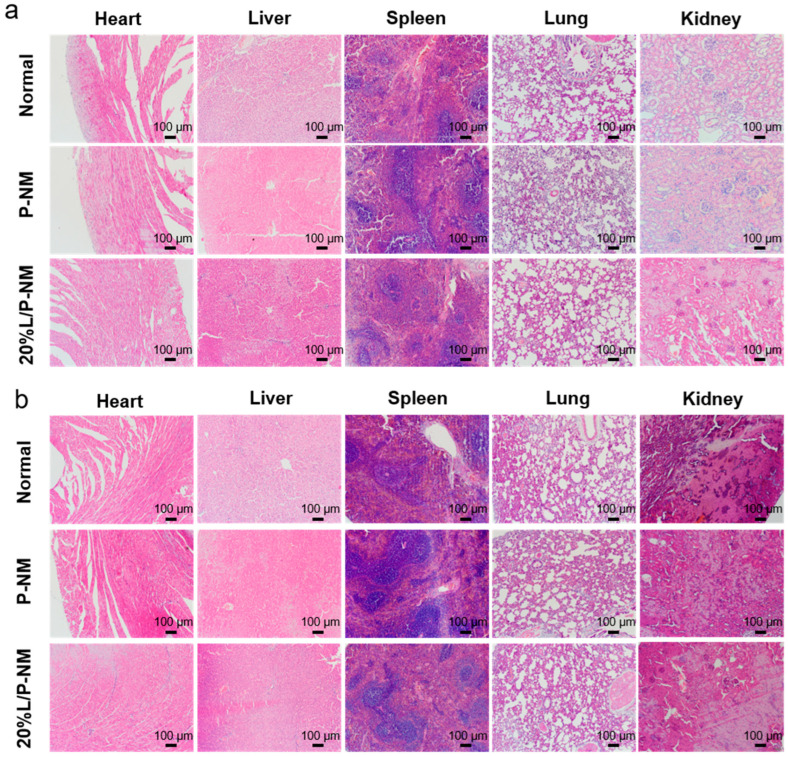
Systemic toxicity evaluation of L/P-NM: (**a**) H&E staining images of the heart, liver, spleen, lungs, and kidneys of the rat abdominal initial adhesion model experiment; (**b**) H&E staining images of the heart, liver, spleen, lungs, and kidneys of the rat abdominal recurrent adhesion model experiment.

To evaluate the systemic toxicity of 20% L/P-NM, we collected and weighed the major organs of each group of rats and performed H&E staining. We also calculated the corresponding organ indices. Upon the comparison of the H&E staining images of the organs in each group (Figure 7b), we found no significant differences between the groups. There was also no significant difference in the organ indices of each group (as shown in Table 1 and Table 2). This indicates that 20% L/P-NM has no systemic toxicity and further validates the biological safety of 20% L/P-NM.

## 4. Conclusions

Starting from the clinical demand for preventing postoperative abdominal adhesion, we have developed a lubricative anti-adhesive nanofibrous membrane based on the hydrated lubrication principle. Our research has found that L/P-NM with various lecithin content exhibits proper cell compatibility and hemocompatibility. The friction test and in vitro anti-cell adhesion test results show that L/P-NM with a 20% lecithin content has the best lubrication and anti-fibroblast adhesion effect. Subsequently, we further evaluated the efficacy of 20%L/P-NM in preventing postoperative abdominal adhesion and recurrent adhesion in an in vivo experiment. The results indicate that 20%L/P-NM can effectively alleviate tissue inflammation and prevent postoperative abdominal adhesion, showing superior anti-adhesive efficacy compared to polylactic acid nanofibrous membranes and also demonstrating the better prevention of more challenging recurrent adhesions. Therefore, as a safe and effective anti-adhesive biomaterial, L/P-NM holds promise for clinical application in the prevention of postoperative abdominal adhesion.

## Figures and Tables

**Table 1:** Index of organs in the rat abdominal initial adhesion model.

Organs	Normal	P-NM	20%L/P-NM
Heart	0.350 ± 0.020	0.354 ± 0.020	0.349 ± 0.022
Liver	3.060 ± 0.281	3.095 ± 0.237	3.026 ± 0.297
Spleen	0.245 ± 0.027	0.231 ± 0.032	0.234 ± 0.024
Lung	0.468 ± 0.021	0.459 ± 0.021	0.467 ± 0.020
Kidney	0.783 ± 0.033	0.794 ± 0.024	0.790 ± 0.030

**Table 2:** Index of organs in the rat abdominal recurrent adhesion model.

Organs	Normal	P-NM	20%L/P-NM
Heart	0.362 ± 0.026	0.359 ± 0.021	0.357 ± 0.022
Liver	3.087 ± 0.221	3.051 ± 0.293	3.126 ± 0.265
Spleen	0.248 ± 0.023	0.245 ± 0.036	0.254 ± 0.025
Lung	0.488 ± 0.018	0.484 ± 0.017	0.493 ± 0.021
Kidney	0.790 ± 0.051	0.795 ± 0.054	0.793 ± 0.037

## Data Availability

Data will be made available on request.

## References

[1] Diamond M.P., Freeman M.L. (2001). Clinical Implications of Postsurgical Adhesions. Hum. Reprod. Update.

[2] Foster D.S., Marshall C.D., Gulati G.S., Chinta M.S., Nguyen A., Salhotra A., Jones R.E., Burcham A., Lerbs T., Cui L. (2020). Elucidating the Fundamental Fibrotic Processes Driving Abdominal Adhesion Formation. Nat. Commun..

[3] Fu S., Yelorda K., Knowlton L. (2021). Are Statins Associated With Reduced Risk of Adhesion-Related Complications After Abdominal Surgery?. JAMA Netw. Open.

[4] Abdiev A., Mamatov N., Toktosunov A., Akeshov A., Kalikiri S. (2022). Results of Repeat Operation for Early Adhesive Intestinal Obstruction. Biomedicine.

[5] Krielen P., Stommel M.W.J., Pargmae P., Bouvy N.D., Bakkum E.A., Ellis H., Parker M.C., Griffiths E.A., Van Goor H., Ten Broek R.P.G. (2020). Adhesion-Related Readmissions after Open and Laparoscopic Surgery: A Retrospective Cohort Study (SCAR Update). Lancet.

[6] Tittel A., Treutner K.H., Titkova S., Öttinger A., Schumpelick V. (2001). New Adhesion Formation after Laparoscopic and Conventional Adhesiolysis: A Comparative Study in the Rabbit. Surg. Endosc..

[7] Catena F., Di Saverio S., Coccolini F., Ansaloni L., De Simone B., Sartelli M., Van Goor H. (2016). Adhesive Small Bowel Adhesions Obstruction: Evolutions in Diagnosis, Management and Prevention?. World J. Gastrointest. Surg..

[8] Sirovy M., Odlozilova S., Kotek J., Zajak J., Paral J. (2024). Current Options for the Prevention of Postoperative Intra-Abdominal Adhesions. Asian J. Surg..

[9] Liao J., Li X., Fan Y. (2023). Prevention Strategies of Postoperative Adhesion in Soft Tissues by Applying Biomaterials: Based on the Mechanisms of Occurrence and Development of Adhesions. Bioact. Mater..

[10] Zhu Y., Zhang C., Liang Y., Shi J., Yu Q., Liu S., Yu D., Liu H. (2024). Advanced Postoperative Tissue Antiadhesive Membranes Enabled with Electrospun Nanofibers. Biomater. Sci..

[11] Wan Z., Wang L., Ma L., Sun Y., Yang X. (2017). Controlled Hydrophobic Biosurface of Bacterial Cellulose Nanofibers through Self-Assembly of Natural Zein Protein. ACS Biomater. Sci. Eng..

[12] Liu C., Zhang X., Zhao L., Hui L., Liu D. (2023). Multilayer Amnion-PCL Nanofibrous Membrane Loaded with Celecoxib Exerts a Therapeutic Effect Against Tendon Adhesion by Improving the Inflammatory Microenvironment. Heliyon.

[13] Raisi A., Dezfoulian O., Davoodi F., Taheri S., Ghahremani S.A. (2021). Salvia Miltiorrhiza Hydroalcoholic Extract Inhibits Postoperative Peritoneal Adhesions in Rats. BMC Complement. Med. Ther..

[14] Giannis D., Geropoulos G., Ziogas I.A., Gitlin J., Oropallo A. (2021). The Anti-adhesive Effect of ANTI-VEGF Agents in Experimental Models: A Systematic Review. Wound Repair Regen..

[15] Mao Y., Chen M., Guidoin R., Li Y., Wang F., Brochu G., Zhang Z., Wang L. (2021). Potential of a Facile Sandwiched Electrospun Scaffold Loaded with Ibuprofen as an Anti-Adhesion Barrier. Mater. Sci. Eng. C.

[16] Kheilnezhad B., Hadjizadeh A. (2022). Ibuprofen-Loaded Electrospun PCL/PEG Nanofibrous Membranes for Preventing Postoperative Abdominal Adhesion. ACS Appl. Bio Mater..

[17] Lan X., Wang H., Bai J., Miao X., Lin Q., Zheng J., Ding S., Li X., Tang Y. (2021). Multidrug-Loaded Electrospun Micro/Nanofibrous Membranes: Fabrication Strategies, Release Behaviors and Applications in Regenerative Medicine. J. Control. Release.

[18] Wang Y., Xu Y., Zhai W., Zhang Z., Liu Y., Cheng S., Zhang H. (2022). In-Situ Growth of Robust Superlubricated Nano-Skin on Electrospun Nanofibers for Post-Operative Adhesion Prevention. Nat. Commun..

[19] Cheng L., Wang Y., Sun G., Wen S., Deng L., Zhang H., Cui W. (2020). Hydration-Enhanced Lubricating Electrospun Nanofibrous Membranes Prevent Tissue Adhesion. Research.

[20] Bednarz S., Wesołowska-Piętak A., Konefał R., Świergosz T. (2018). Persulfate Initiated Free-Radical Polymerization of Itaconic Acid: Kinetics, End-Groups and Side Products. Eur. Polym. J..

[21] Jiang X., Han J., Cao L., Bao Y., Shi J., Zhang J., Ni L., Chen J. (2017). A Facial Strategy for Catalyst and Reducing Agent Synchronous Separation for AGET ATRP Using Thiol-Grafted Cellulose Paper as Reducing Agent. Polymers.

[22] Zhang Q., Yao D., Rao B., Jian L., Chen Y., Hu K., Xia Y., Li S., Shen Y., Qin A. (2021). The Structural Basis for the Phospholipid Remodeling by Lysophosphatidylcholine Acyltransferase 3. Nat. Commun..

[23] Morita S., Ikeda Y. (2022). Regulation of Membrane Phospholipid Biosynthesis in Mammalian Cells. Biochem. Pharmacol..

[24] Richey Levine A., Picoraro J.A., Dorfzaun S., LeLeiko N.S. (2022). Emulsifiers and Intestinal Health: An Introduction. J. Pediatr. Gastroenterol. Nutr..

[25] Mortensen A., Aguilar F., Crebelli R., Di Domenico A., Frutos M.J., Galtier P., Gott D., Gundert-Remy U., Lambré C., EFSA Panel on Food Additives and Nutrient Sources Added to Food (ANS) (2017). Re-Evaluation of Lecithins (E 322) as a Food Additive. EFSA J..

[26] Płaczek M., Wątróbska-Świetlikowska D., Stefanowicz-Hajduk J., Drechsler M., Ochocka J.R., Sznitowska M. (2019). Comparison of the in Vitro Cytotoxicity among Phospholipid-Based Parenteral Drug Delivery Systems: Emulsions, Liposomes and Aqueous Lecithin Dispersions (WLDs). Eur. J. Pharm. Sci..

[27] Bot F., Cossuta D., O’Mahony J.A. (2021). Inter-Relationships between Composition, Physicochemical Properties and Functionality of Lecithin Ingredients. Trends Food Sci. Technol..

[28] Li G., Zhao M., Xu F., Yang B., Li X., Meng X., Teng L., Sun F., Li Y. (2020). Synthesis and Biological Application of Polylactic Acid. Molecules.

[29] Dong Y., Kampf N., Schilt Y., Cao W., Raviv U., Klein J. (2022). Dehydration Does Not Affect Lipid-Based Hydration Lubrication. Nanoscale.

[30] Klein J. (2013). Hydration Lubrication. Friction.

[31] Liu X., Wang H., She J., Zhang Q., Hu Q., Li D., Wu H., Ye X., Diao R., Shi X. (2023). An Anti-Fibroblast Adhesion and Anti-Inflammatory Hydrogel Film Combined with VEGF for Intrauterine Adhesion Prevention. Chem. Eng. J..

[32] Shalumon K.T., Sheu C., Chen C.-H., Chen S.-H., Jose G., Kuo C.-Y., Chen J.-P. (2018). Multi-Functional Electrospun Antibacterial Core-Shell Nanofibrous Membranes for Prolonged Prevention of Post-Surgical Tendon Adhesion and Inflammation. Acta Biomater..

[33] Mao Y., Zeng Y., Meng Y., Li Y., Wang L. (2022). GelMA and Aliphatic Polyesters Janus Nanofibrous Membrane with Lubrication/Anti-Fibroblast Barrier Functions for Abdominal Adhesion Prevention. Eur. Polym. J..

[34] Shalem A., Yehezkeli O., Fishman A. (2024). Enzymatic Degradation of Polylactic Acid (PLA). Appl. Microbiol. Biotechnol..

[35] Qi X., Ren Y., Wang X. (2017). New Advances in the Biodegradation of Poly(lactic) Acid. Int. Biodeterior. Biodegrad..

[36] Meng L., Liu X., Liu L., Hong Q., Cheng Y., Gao F., Chen J., Zhang Q., Pan C. (2022). Comparative Investigation of the Corrosion Behavior and Biocompatibility of the Different Chemical Conversion Coatings on the Magnesium Alloy Surfaces. Metals.

[37] Ruiz-Esparza G.U., Wang X., Zhang X., Jimenez-Vazquez S., Diaz-Gomez L., Lavoie A.-M., Afewerki S., Fuentes-Baldemar A.A., Parra-Saldivar R., Jiang N. (2021). Nanoengineered Shear-Thinning Hydrogel Barrier for Preventing Postoperative Abdominal Adhesions. Nano-Micro Lett..

[38] Erdi M., Saruwatari M.S., Rozyyev S., Acha C., Ayyub O.B., Sandler A.D., Kofinas P. (2023). Controlled Release of a Therapeutic Peptide in Sprayable Surgical Sealant for Prevention of Postoperative Abdominal Adhesions. ACS Appl. Mater. Interfaces.

[39] Helmedag M.J., Heise D., Eickhoff R.M., Schmitz S.M., Mechelinck M., Emonts C., Bolle T., Gries T., Neumann U.P., Klink C.D. (2022). Ultra-Fine Polyethylene Hernia Meshes Improve Biocompatibility and Reduce Intraperitoneal Adhesions in IPOM Position in Animal Models. Biomedicines.

[40] Wang R., Guo T., Li J. (2022). Mechanisms of Peritoneal Mesothelial Cells in Peritoneal Adhesion. Biomolecules.

[41] Pitenis A.A., Urueña J.M., Hart S.M., O’Bryan C.S., Marshall S.L., Levings P.P., Angelini T.E., Sawyer W.G. (2018). Friction-Induced Inflammation. Tribol. Lett..

